# Mitochondrial Diversity and Distribution of African Green Monkeys (*Chlorocebus* Gray, 1870)

**DOI:** 10.1002/ajp.22113

**Published:** 2013-01-10

**Authors:** Tanja Haus, Emmanuel Akom, Bernard Agwanda, Michael Hofreiter, Christian Roos, Dietmar Zinner

**Affiliations:** 1 Cognitive Ethology Laboratory, German Primate Center, Leibniz Institute for Primate ResearchGoettingen, Germany; 2 Primate Genetics Laboratory, German Primate Center, Leibniz Institute for Primate ResearchGoettingen, Germany; 3 Centre of Biodiversity and Sustainable Land Use, University of GoettingenGoettingen, Germany; 4 College of Agriculture and Natural Resources, Kwame Nkrumah University of Science and TechnologyKumasi, Ghana; 5 Mammalogy Section, National Museums of KenyaNairobi, Kenya; 6 Department of Biology, University of YorkYork, United Kingdom; 7 Gene Bank of Primates, German Primate Center, Leibniz Institute for Primate ResearchGoettingen, Germany

**Keywords:** cytochrome *b* gene, phylogeny, hybridization, introgression, African savanna

## Abstract

African green monkeys (*Chlorocebus*) represent a widely distributed and morphologically diverse primate genus in sub-Saharan Africa. Little attention has been paid to their genetic diversity and phylogeny. Based on morphological data, six species are currently recognized, but their taxonomy remains disputed. Here, we aim to characterize the mitochondrial (mt) DNA diversity, biogeography and phylogeny of African green monkeys. We analyzed the complete mitochondrial cytochrome *b* gene of 126 samples using feces from wild individuals and material from zoo and museum specimens with clear geographical provenance, including several type specimens. We found evidence for nine major mtDNA clades that reflect geographic distributions rather than taxa, implying that the mtDNA diversity of African green monkeys does not conform to existing taxonomic classifications. Phylogenetic relationships among clades could not be resolved suggesting a rapid early divergence of lineages. Several discordances between mtDNA and phenotype indicate that hybridization may have occurred in contact zones among species, including the threatened Bale monkey (*Chlorocebus djamdjamensis*). Our results provide both valuable data on African green monkeys’ genetic diversity and evolution and a basis for further molecular studies on this genus. Am. J. Primatol. 75:350-360, 2013. © 2013 Wiley Periodicals, Inc.

## INTRODUCTION

**Fig. 1:**
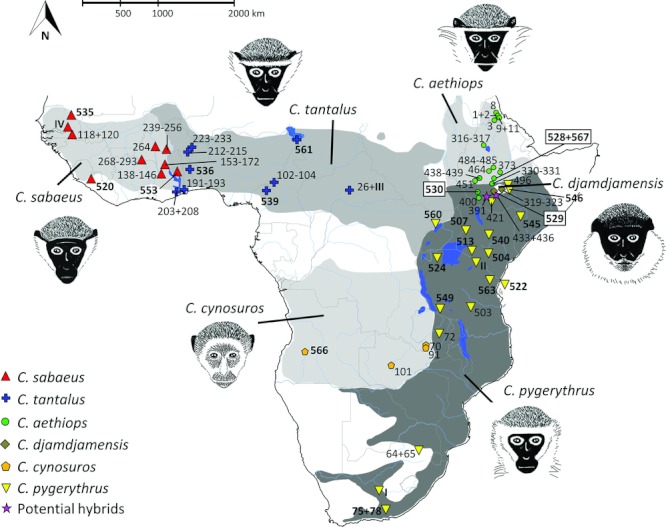
Distribution of African green monkeys (*Chlorocebus*) and collection sites of fecal and museum (bold) samples. Species distributions are shaded and modified from Lernould (1988) and Kingdon (1997). Colored symbols indicate phenotypes determined. Numbers correspond to IDs in Fig. 2 and Supporting Information Table SI. IDs of type specimens are boxed. Schematic drawings depicting main differences in facial characters are redrawn from Hill (1966).

African green monkeys of the genus *Chlorocebus* occur in savanna habitats across sub-Saharan Africa (Fig. 1) [Kingdon, 1997; Lernould, 1988]. Previously, African green monkeys have been subsumed into the *aethiops* group of the genus *Cercopithecus* [Dandelot, 1971; Grubb et al., 2003; Hill, 1966; Napier, 1981; Schwarz, 1926], but based on recent morphological and genetic studies, they are now separated from *Cercopithecus* and placed within the genus *Chlorocebus* as sister taxon to the other ground dwelling members (*Erythrocebus, Allochrocebus*) of the Cercopithecini [Groves, 2001, 2005; Mekonnen et al., 2010a, 2010bb; Perelman et al., 2011; Tosi et al., 2002; Xing et al., 2007; but see Grubb et al. 2003 for a different opinion]. Due to their wide distribution and phenotypic diversity, 22 taxa have been described with most of them now being recognized as synonyms [Dandelot, 1971; Groves, 2001; Hill, 1966; Napier, 1981; Schwarz, 1926]. However, their taxonomy is still disputed and some researchers consider *Chlorocebus aethiops* as one polytypic species comprising five or six subspecies [Elton et al., 2010; Grubb et al., 2003; Kingdon, 1997], whereas Dandelot [1971] preferred a classification with four species and several subspecies. Here, we follow the taxonomy of Groves [2001, 2005] as his classification combines the most recent findings on genetics, morphology, and ecology on generic as well as on species and subspecies level. He recognizes six species that is also followed by the IUCN red list of threatened species [IUCN, 2012]; four monotypic species: *C. aethiops* (grivet), *C. djamdjamensis* (Bale monkey), *C. sabaeus* (green monkey), and *C. cynosuros* (malbrouck monkey), and two polytypic species: *C. tantalus* (tantalus monkey) with subspecies *C. tantalus budgetti*, *C. t. marrensis* and *C. t. tantalus*, and *C. pygerythrus* (vervet) with subspecies *C. pygerythrus hilgerti*, *C. p. excubitor*, *C. p. nesiotes*, *C. p. rufoviridis*, and *C. p. pygerythrus*.

Whereas most species inhabit wide geographic ranges and are listed as “Least Concern” in the IUCN red list of threatened species [Butynski, 2008; Kingdon & Butynski, 2008; Kingdon & Gippoliti, 2008a,b; Kingdon et al., 2008], *C. djamdjamensis* is endemic to the highlands of South Ethiopia and is classified as “Vulnerable” [Butynski et al., [Bibr b8]]. In addition to ongoing habitat disturbance, Kingdon [1997] assumed that *C. djamdjamensis* is additionally threatened by hybridization with the lowland forms *C. aethiops* and *C. pygerythrus*. Hybridization seems to be not uncommon within *Chlorocebus* as it has been reported from most species contact zones in East Africa, and even intergeneric hybridization with *Cercopithecus mitis* has been observed [deJong & Butynski 2010; Kingdon, 1997; Mekonnen et al., 2012; Napier, 1981]. However, with respect to *C. djamdjamensis*, both its taxonomic status and the potential threat by hybridization remain unclear without genetic analyses.

Whatever taxonomic classification is applied to the members of *Chlorocebus*, the phylogenetic relationships among the different taxa are unresolved and a comprehensive phylogenetic analysis has yet to be done [Groves, 2001; Grubb et al., 2003]. Even though African green monkeys are used as model organism in biomedical research, for example, in Simian Immunodeficiency Virus (SIV) research [Switzer et al., 2004, 2005; Wertheim & Worobey, 2007], only few intrageneric genetic studies have been conducted, either focusing on a small region in Ethiopia [Shimada, 2000; Shimada et al., 2002] or relying on only small and taxonomically incomplete data sets [van der Kuyl et al., 1995, 1996]. Based on this data, the origin of green monkeys from the Caribbean islands (Barbados, St. Kitts, and Nevis), which are widely used in biomedical research, was assumed to be in Senegal or Gambia [van der Kuyl et al., 1996]. Although complete mitochondrial genomes were analyzed in a study on co-evolutionary processes between African green monkeys and their host-specific SIVs [Wertheim & Worobey, 2007], a total of just six individuals of four taxa representing only a small part of the genus’ geographical range were included in this study.

Given the conflicting taxonomic classifications and the lack of genetic analyses covering all major *Chlorocebus* taxa and the complete geographical range of the genus, we attempt to clarify the genetic diversity of African green monkeys using a comprehensive data set representing all species. In the present study, we analyzed the complete mitochondrial cytochrome *b* (cyt *b*) gene from 126 African green monkey samples with the aim to evaluate the mitochondrial diversity within the genus, to delineate geographic ranges of taxa, and to elucidate their phylogenetic relationships.

## METHODS

### Sample Collection

We collected 91 fecal samples of wild African green monkeys originating from 32 sites in Senegal, Ghana, Burkina Faso, Nigeria, Ethiopia, Zambia, Tanzania, and the Republic of South Africa (RSA) sampled between 2005 and 2010 (Fig. 1, Supporting Information Table SI). Samples were kept for at least 24 hr in >90% ethanol and, after drying, stored on silica beads [Nsubuga et al., 2004]. Only few samples from Nigeria and Zambia were stored directly on silica (dry samples) or only in ethanol (fresh samples). We determined geographic coordinates of sample localities using GPS (Supporting Information Table SI). We further included seven hair samples from zoos and 24 museum samples (skin, dried soft tissue, or teeth, Supporting Information Table SI), including samples of holotypes of *djamdjamensis* (sample ID 529), *ellenbecki* (sample ID 528), and *matschiei* (sample ID 530), and a paratype of *ellenbecki* (sample ID 567, Fig. 1). We used only museum samples with clear provenance. As there is no detailed information on the origin of the hair sample from the Central African Republic (CAR, ID 26), we depicted the locality in the center of the country (Fig. 1). For museum samples, we used approximate coordinates of sampling sites based on voucher localities. We complemented our sample set with already published sequences of four individuals from Senegal, Tanzania/Kenya, CAR, and RSA [Wertheim & Worobey, 2007]. As the exact origins of these individuals are unknown, we depicted approximate sample localities in the map according to van der Kuyl et al. [1996] (I-IV in Fig. 1).

For the determination of species (according to the taxonomy of Groves [2001]) in the field and of museum specimens, we applied chief distinguishing phenotypic characters by direct observation [Groves, 2001; Hill, 1966; Napier, 1981]. For facial pattern, we recorded in particular information on color and structure of whiskers and the white frontal band as well as on the presence of the white mustache (Fig. 1). Further, we used the presence of the paracaudal white tuft and the subcaudal red patch, and information on the color of extremities and tail tips. As we do not have phenotypic information on analyzed specimens from Zambia, CAR, and GenBank, we assigned them to species according to their geographical provenance. In total, our data set comprises 126 samples from 59 sites representing all six proposed *Chlorocebus* species (Fig. 1).

All research in this project complied with protocols approved by the German Primate Center in Germany, Ethiopian Wildlife and Conservation Authority (EWCA) in Ethiopia, the Centre National de la Recherche Scientifique et Technologique (CNRST) in Burkina Faso, the Forestry Commission (FC) of Ghana, and the National Museums of Kenya (NMK) in Kenya, and adhered to the legal requirements of the countries in which the research was conducted. The study was carried out in compliance with respective animal care regulations and the principles of the American Society of Primatologists for the ethical treatment of non-human primates.

### Extraction, Amplification, and Sequencing of DNA

Extraction of total genomic DNA from fecal samples was performed with the QIAamp DNA Stool Mini Kit (Qiagen, Germany) following standard protocols with only minor changes. Samples were incubated in ASL buffer overnight and DNA was eluted in 210 μl water (high-performance liquid chromatography (HPLC) grade) instead of AE buffer. The extracts were stored in 50 μl aliquots at –20°C for up to 24 months before further processing. For analysis of hair samples from zoos, roots of three to five hairs were directly added to the polymerase chain reaction (PCR) mix without prior DNA extraction [Fontanesi et al., 2007; Roos et al., 2008]. For the extraction of museum samples (teeth, pelt/skin, dried soft tissue), we used a Guanidinium thiocyanate (GuSCN) buffer (5 M GuSCN, 25 mM NaCl, 50 mM Tris, 20 mM EDTA, 1% Tween 20, 1% beta-mercaptoethanol) modified from Rohland et al. [2004]. Samples were incubated for about 24 hr in 1 ml extraction buffer per 50 mg sample under constant agitation at room temperature in the dark. We purified DNA with a combination of a batch-based silica and a column-based method according to Rohland and Hofreiter [2007] and Rohland et al. [2010], and eluted the DNA in 50 μl TE buffer. To avoid contamination with modern DNA, extractions of museum samples were conducted in a laboratory dedicated to ancient DNA analysis at the University of York. To monitor for possible cross-sample contamination, we performed one to three blank extractions (without sample) per extraction depending on the number of samples processed.

Since in mammals with female philopatry, mtDNA is known to conserve geographical pattern better than nuclear DNA [Avise, 2009], we analyzed the mitochondrial cyt *b* gene, which has been successfully used to resolve phylogenetic relationships in several mammals [Agnarsson & May-Collado, 2008; Castresana, 2001; Roos et al., 2008; Tobe et al., 2010; Van Ngoc Thinh et al., 2010]. We amplified the complete cyt *b* (1,140 bp) gene via two or four (fecal samples), or even six (museum samples) overlapping fragments (Supporting Information Table SII), because most of our samples are expected to yield only degraded DNA. For hair samples only, we used a nested PCR approach with external primers first, and subsequently, with primers amplifying two overlapping fragments in separate PCR reactions. We used 1 U BiothermTaq 5000 (Genecraft, Germany) for hair and fecal samples in a 30 μl PCR mix (1× reaction buffer, 0.16 mM for each dNTP, 0.33 μM for each primer, and 0.6 mg/ml BSA), with the following thermo cycler conditions: 94°C for 2 min, followed by 40 cycles of 94°C for 1 min, 62°C for 1 min, 72°C for 1 min, and 72°C for 5 min. For the amplification of museum samples, we used 3 U AmpliTaq Gold 360 (Applied Biosystems, Germany) in a 20 μl mix (1× reaction buffer, 2 mM MgCl_2_, 0.25 mM for each dNTP, 0.75 μM for each primer, and 0.1 mg/ml BSA) and the following PCR conditions: 94°C for 10 min, followed by 60 cycles of 94°C for 30 sec, 62°C for 45 sec, 72°C 45 sec, and 72°C for 5 min. To test for reliability of sequences generated from museum samples, we randomly replicated at least two of the six cyt *b* fragments for each sample. For 11 museum samples, for which we found putative nuclear insertions of mitochondrial sequences (NUMTs) in one or two of the six fragments, we amplified longer fragments (up to 555 bp). PCR reactions were conducted with one or two PCR blanks (HPLC-purified water) in addition to the extraction blanks depending on the number of samples processed. We ran all PCR products on 1–2% agarose gels and, after excision, purified PCR products with the Qiagen Gel Extraction Kit (Qiagen, Germany). Subsequently, sequences were run on an ABI 3130*xL* sequencer using the BigDye Terminator Cycle Sequencing Kit (Applied Biosystems, Germany) and respective forward and reverse primers. All sequences were deposited in GenBank (for GenBank accession numbers see Supporting Information Table SI).

### Statistical Analyses

We assembled and aligned sequences with the program Geneious Pro 5.0.4 [Drummond et al., 2011] and corrected them by eye. To check for the presence of NUMTs, simple neighbor-joining trees for gene fragments were reconstructed in Mega 5.0 [Tamura et al., 2011] and branch lengths and depicted relationships were visually checked to be similar. Furthermore, all sequences were translated into amino acid sequences to detect unexpected stop codons.

We applied maximum-likelihood (ML) and Bayesian approaches for phylogenetic tree reconstructions using the programs Garli 2.0 [Zwickl, 2006] and MrBayes 3.1.2 [Huelsenbeck et al., 2001; Ronquist & Huelsenbeck, 2003]. For tree reconstructions, we used only unique sequences; therefore, the final alignment included 68 haplotypes of African green monkeys and one ortholog of *Erythrocebus patas* used as outgroup. For both reconstructions, the appropriate model of nucleotide substitution (TrN + G) was chosen according to the Bayesian Information Criterion as implemented in jModeltest 0.1 [Guindon & Gascuel, 2003; Posada, 2008]. For the ML analysis, support of internal nodes was assessed by 500 bootstrap replications in four independent runs. All other settings were left at their default value. A 50% majority-rule consensus tree was calculated with Paup* 4b10 [Swofford, 2003]. For Bayesian reconstructions, we applied 10 million generations with tree and parameter sampling every 10,000 generations. We checked the output of MrBayes for the adequacy of effective sample size values and discarded the first 25% of sampled trees and parameters from the beginning of the chain as burn in.

We used Network 4.610 [Bandelt et al., 1999] to additionally explore biogeographic patterns and to compare patterns of phenotype and mtDNA. We calculated a median-joining network based on cyt *b* sequences of the complete data set including all 126 samples. Results were displayed and edited using the Network Publisher software. To compare intra- and interspecific distances of obtained mtDNA clades or lineages, we used the software Mega 5.0 [Tamura et al., 2011]. We calculated the number of substitutions per site between sequences with the Tamura-Nei model and a gamma distribution.

## RESULTS

We successfully amplified and sequenced the complete cyt *b* gene from 122 samples. Together with four sequences from GenBank, the data set comprised 126 African green monkey sequences. Among them, we detected 68 unique haplotypes, which are characterized by a total of 329 variable sites of which 226 are parsimony informative.

Based on directly observed phenotypic characters, we clearly assigned samples from all regions to one of the six recognized *Chlorocebus* species (Fig. 1), except of some samples from Ethiopia (pink stars in Fig. 1), where we found phenotypes showing mixed characters of *C. aethiops* and *C. djamdjamensis* (sample IDs 433 and 436) or phenotypes of both *C. aethiops* and *C. pygerythrus* within one group (sample ID 391).

**Fig. 2:**
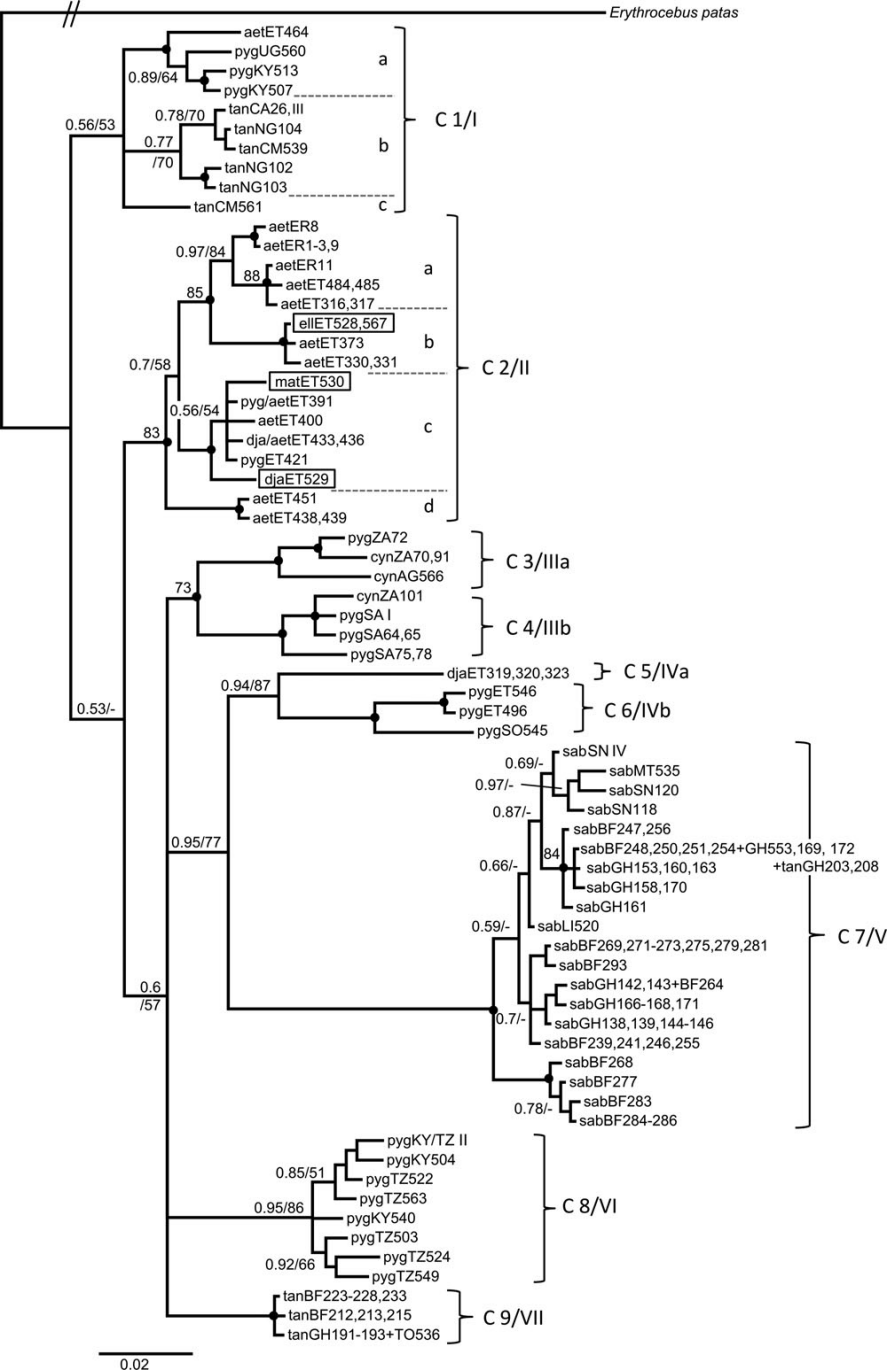
Bayesian phylogram with posterior probabilities and ML bootstrap support values based on the complete cyt *b* gene. C1/I-C9/VII indicate main mtDNA clades. Bootstrap support values of >90% and posterior probabilities of >0.98 are presented as black dots; values below are given at respective nodes. Type specimens are boxed.

**Fig. 3:**
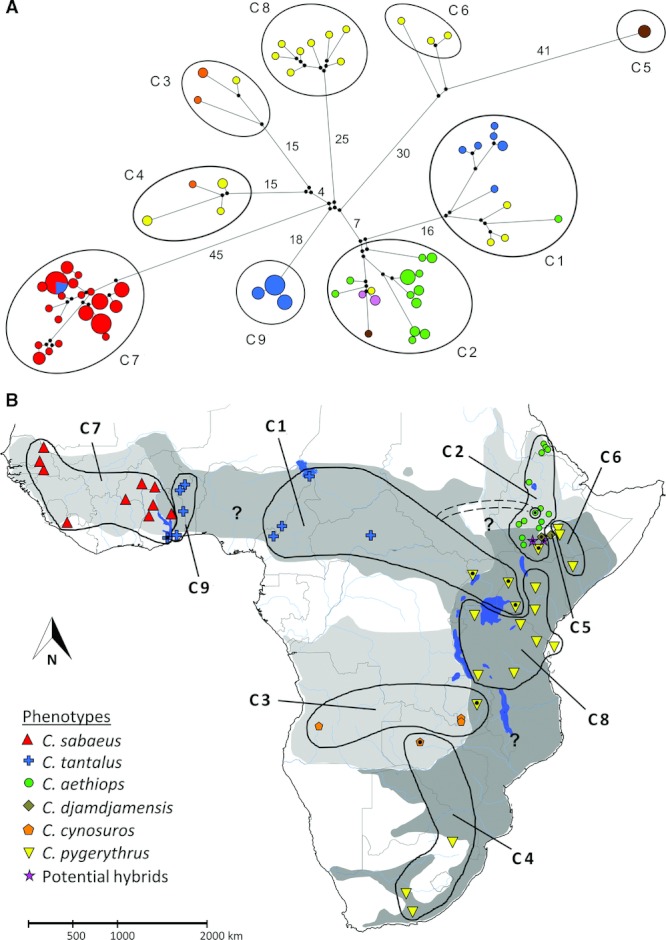
(A) Median-joining network of mtDNA sequences with depicted clade affiliations (see Fig. 2). Sizes of circles indicate haplotype frequencies and colors represent different phenotypes. Black dots along branches represent median vectors and branch length is relative to the number of mutated positions. (B) Map showing geographic distribution of the mtDNA clades detected. Samples indicating discordance between mtDNA and observed phenotype are highlighted with black dots. Question marks indicate recommended regions for future studies.

ML and Bayesian tree reconstructions resulted in nearly identical tree topologies showing seven or nine major mtDNA clades or lineages (C1/I-C9/VII, hereafter clades, Fig. 2). Monophyly of several clades is not strongly supported and these clades might be further divided into several subclades (C1/I a-c, C2/II a-d, C IIIa-b, and C IVa-b, respectively, Fig. 2). We additionally examined genetic differences within and among clades, which show profound overlap of genetic distances in C III and C IV when determining only seven clades supporting a division into nine major mtDNA clades (Supporting Information Fig. S1). In the median-joining network, the same nine clades became apparent revealing good correspondence to geographic regions, except for the *C. aethiops* sample from Ethiopia, which falls together with samples from Nigeria, Cameroon, CAR, Uganda, and Kenya into C1 (Fig. 3). Phylogenetic relationships among clades remained largely unresolved due to low statistical support of both ML and Bayesian approaches (Fig. 2).

The nine major clades comprise the following species and type specimens as delineated by phenotypes and geographic regions: C1—*C. tantalus* from Nigeria and CAR, *C. aethiops* from Woliso in Ethiopia, and *C. pygerythrus* from Uganda and Kenya; C2—*C. aethiops* from Ethiopia and Eritrea, the type specimens of *ellenbecki* and *matschiei*, *C. djamdjamensis* from Bubbe Kersa and Gossa, the holotype of *djamdjamensis* from Abera, and *C. pygerythrus* from Yabello in South Ethiopia; C3—*C. cynosuros* from Angola and Northwest Zambia, and *C. pygerythrus* from Northeast Zambia; C4—*C. pygerythrus* from South Africa, and *C. cynosuros* from South Zambia; C5—*C. djamdjamensis* from the Bale Mountains National Park (NP) in Ethiopia; C6—*C. pygerythrus* east of the Bale Mountains in Ethiopia and Somalia; C7—*C. sabaeus* from West Africa west of the Volta and Oti River and *C. tantalus* from Shai Hills Resource Reserve (West of the Volta River); C8—*C. pygerythrus* from Kenya and Tanzania; and C9—*C. tantalus* from east of the Volta and the Oti River in Ghana, Burkina Faso, and Togo (Figs. 2 and 3). With exception of *C. sabaeus* (C7), phenotypes of all species are found in more than one major clade due to discordance between phenotype and mtDNA (*C. tantalus*: C1, C7, C9; *C. aethiops*: C1, C2; *C. cynosuros*: C3, C4; *C. pygerythrus*: C1, C2, C3, C4, C6, C8; *C. djamdjamensis*: C2, C5) causing several instances of paraphyly within the genus *Chlorocebus* (Figs. 2 and 3).

## DISCUSSION

Our results show that the mtDNA diversity does not conform to existing taxonomic classifications [Dandelot 1959, 1971; Groves, 2001, 2005; Hill, 1966; Kingdon, 1997; Lernould, 1988; Napier, 1981], neither if we apply a six species classification nor a one superspecies classification. Furthermore, several discordances between phenotype and mtDNA, which are exclusively found in samples from regions close to contact zones among species (Fig. 3), point to possible hybridization. Hybridization and consequential discordance between mtDNA and nuclear DNA is a common pattern in cercopithecines [Detwiler et al., 2005; Keller et al., 2010; Zinner et al., 2009a,b, 2011]. Since pheno- or morphotypes are more likely to be consistent with nuclear than with mtDNA phylogenies [Zinner et al., 2009a], we assume that introgressive hybridization is responsible for the discordances in our phylogeny of African green monkeys and that intrageneric gene flow is common among all *Chlorocebus* species. Thereby, introgression would not vanish if a subspecies taxonomy is applied. The exchange of genetic information from one taxon to another would remain, either between species or subspecies.

Based on phylogenetic tree reconstructions, we distinguish either seven or nine major mtDNA clades. However, based on the comparison of genetic distances, the division into nine clades shows no overlap of inter- and intraclade distances and seems to be more appropriate. Both assignments reflect geographic regions rather than nominal species (Figs. 2 and 3). Monophyly of most clades is not well supported and irrespective of the number of clades, our results clearly show that cyt *b* sequence information does not allow any taxonomic inferences. However, based on descriptions of respective holotypes [Groves, 2001; Napier, 1981; Schwarz, 1926], the nine major mtDNA clades fall into the geographic range of the following taxa: C1 = *C. tantalus*, C2 = *C. aethiops*, C3 = *C. cynosuros*, C4 = *C. p. pygerythrus*, C5 = *C. djamdjamensis*, C6 = *C. p. hilgerti*, C7 = *C. sabaeus*, and C8 = *C. p. rufoviridis*. Since for *C. tantalus*, a type locality is not available and no taxon has been described from the region of Ghana, Burkina Faso, or Togo, the western phenotypic *tantalus* clade (C9) cannot be referred to the geographic range of any previously described taxon. However, Schwarz [1926] already mentioned a phenotypically different form from Togo that remained undescribed and was not recognized by others [e.g., Booth, 1956; Hill, 1966].

While the holotype of *matschiei* has been phenotypically assigned to *C. aethiops* by most authors [Groves, 2001; Hill, 1966; Napier, 1981], type specimens of *ellenbecki* have been either referred to representatives of *C. pygerythrus* [Groves, 2001; Napier, 1981] or *C. aethiops* [Dandelot and Prevost, 1972; Hill, 1966]. Based on observed phenotypic characters, we assigned type specimens of both *ellenbecki* (sample IDs 528 and 567) and *matschiei* (sample ID 530) to *C. aethiops* (Fig. 1), which is supported by our mtDNA results. Therefore, both taxa might represent synonyms of *C. aethiops* based on our findings. Schwarz [1926] explained the distinct characters of the holotype of *C. djamdjamensis* with a local adaption to the harsh mountain climate and listed it as synonym for *hilgerti*. Although we found the main distinguishing features of the holotype to be characteristic of *C. djamdjamensis*, the haplotype of the holotype falls into the *C. aethiops* clade and does not cluster with the distinct *C. djamdjamensis* lineage from the Bale Mountains National Park. This indicates that the holotype of *djamdjamensis* possibly represents a hybrid between *C. djamdjamensis* and *C. aethiops*.

Based on a previous study, *C. sabaeus* from St. Kitts originates most probably from Senegal or Gambia [van der Kuyl et al., 1996]. In our study, we included the cyt *b* sequence of the same reference individual that was used in the study by van der Kuyl et al. [1996, sample ID IV, Supporting Information Table SI], and found that this sample clusters together with other samples from Senegal and Mauretania and are not intermingled with samples from Ghana and Burkina Faso (Fig. 2). Therefore our data support the hypothesis that Caribbean green monkeys originate from Senegal or adjacent countries of the West African coast.

Compared to previous reports, we found some differences in geographic positions of species borders and contact zones. In West Africa, Booth [1956] described that the border between *C. sabaeus* and *C. tantalus* follows the Volta and the White Volta River in Ghana. We found the easternmost sample of *C. sabaeus* in Krachi, which is east of the White Volta River (sample ID 553) in Ghana. As for this sample, there is no discordance between phenotype and mtDNA, we assume that the border between *C. sabaeus* (C7) and the western *tantalus* clade (C9) possibly follows the Oti River and not the White Volta River in Ghana and Burkina Faso. Several authors mentioned an exceptional *C. tantalus* population west of the Volta River on the Accra plains in South Ghana [Booth, 1956, 1958; Hill, 1966; Kingdon, 1997; Napier, 1981], which could be confirmed in our study based on phenotypic data. However, the mtDNA sequences of these *C. tantalus* individuals fall into the *C. sabaeus* clade (C7, Fig. 3). Since no individuals with *sabaeus* phenotypes have been found in this area, historic introgressive hybridization is the most probable explanation for the discordance between phenotype and mtDNA of the *C. tantalus* population. Concerning the *C. tantalus* clade in Ghana, Burkina Faso, and Togo (C9), future studies should consider samples from Benin and Nigeria, especially east and west of the Niger River, to delimitate the geographical range of this western *C. tantalus* clade and to test if the mtDNA border between the western (C9) and eastern *C. tantalus* (C1) clades follows the Niger River (Fig. 3).

In Ethiopia, mtDNA of phenotypes from all three species *C. aethiops*, *C. djamdjamensis*, and *C. pygerythrus* cluster together in the clade from South Ethiopia (C2, Fig. 3), which indicates that hybridization has occurred and possibly still occurs between all three species in South Ethiopia. Groves [2001] mentioned a possible boundary between *C. aethiops* and *C. pygerythrus* between Lake Shala and Lake Zwai. Our phenotypic data provide evidence that the contact zone between *C. aethiops* and *C. pygerythrus* is about 200 km further to the south close to Lake Abaya, because we found phenotypes of *C. aethiops* and *C. pygerythrus* as well as intermediate forms in this area (sample ID 391, Fig. 1), and no phenotypes of *C. pygerythrus* further to the north. Based on phenotypic characters, we suggest that individuals from Bubbe Kersa and Gossa (sample IDs 433 and 436) are potential hybrids between *C. djamdjamensis* and *C. aethiops*. MtDNA of samples from those individuals cluster in the *aethiops* clade (C2), which supports the assumption that hybridization occurs between *C. aethiops* and *C. djamdjamensis* in this area. Interestingly, mtDNA of the holotype of *djamdjamensis* (sample ID 529), which was collected close to Bubbe Kersa and Gossa in Abera in 1900, also represents a putative hybrid (Figs. 2 and 3). These results do not only provide evidence for ongoing hybridization among *C. aethiops* and *C. djamdjamensis*, but also indicate that hybridization already occurred more than 100 years ago in this area. In concordance with Shimada [2000], samples of *C. aethiops* from Woliso in Southwest Ethiopia cluster together with samples of *C. pygerythrus* from Uganda and Kenya in the *C. tantalus* clade (C1, Figs. 2 and 3). Since we did not observe *C. tantalus* phenotypes in Ethiopia, ongoing hybridization between *C. aethiops* and *C. tantalus* in this area is unlikely and has not been reported yet. The White Nile River was mentioned as possible barrier between *C. aethiops* and *C. tantalus*, but no reliable data about the distribution of *C. aethiops* and *C. tantalus* in this region is available [Engelberger, 2010; Lernould, 1988] (Fig. 3). We found further indication for hybridization between the *C. p. rufoviridis* (C8) and the *C. tantalus* (C1) clade in Uganda and Kenya, as we detected phenotypes of *C. pygerythrus* but mtDNA of *C. tantalus* in this region (C1, Fig. 3). Our findings confirm Napier's [1981] assumption of hybridization between *C. tantalus*, *C. pygerythrus*, *C. aethiops*, and *C. djamdjamensis* in East Africa, who assumed a broad hybrid zone spreading from Uganda northeastwards to Harar in Ethiopia.

Since we do not have information on phenotypes of individuals from Zambia, we cannot exclude that incongruences within *C. cynosuros* and *C. pygerythrus* are simply caused by wrong taxonomic determination of the specimens. Thus, *C. pygerythrus* may be distributed further to the North and *C. cynosuros* further to the east than previously believed (Fig. 3). Denser sampling of *C. pygerythrus* and *C. cynosuros* in Zambia is needed to delineate their geographical ranges and to study whether interspecific gene flow occurs in a respective contact zone. Furthermore additional samples from Southern Africa would help to clarify paraphyletic relationships within the widely distributed species *C. pygerythrus* stretching from Ethiopia to South Africa.

The analysis of the complete cyt *b* gene has been successfully used to reveal the phylogeny of several primates and mammals in general [Agnarsson and May-Collado, 2008; Castresana, 2001; Roos et al., 2008; Tobe et al., 2010; Van Ngoc Thinh et al., 2010]. This was not possible for *Chlorocebus*. Although analyses revealed several mtDNA clades, we were not able to resolve phylogenetic relationships within the genus. While additional samples from Southern Africa might contribute to a better resolution of phylogenetic relationships within African green monkeys, weak statistical support and consequential uncertainties in basal relationships might also be an indication for the divergence of main lineages within a short time period [Zinner et al., 2009a]. Recurrent gene flow among parapatric species, triggered by periodic retractions and expansions of populations in response to Pleistocene climate changes, is another possible reason for the ambiguous relationships, as it has been found in several African savanna mammals including primates (e.g., *Papio*) [Arctander et al., 1999; Flagstad et al., 2001; Lorenzen et al., 2007; Muwanika et al., 2003; Zinner et al., 2009a]. The appearing of nine mtDNA clades at least indicates that *Chlorocebus* has experienced certain periods of geographic isolation in its evolutionary history. However, to test if the phylogeography of African green monkeys has been influenced by Pleistocene climate oscillations as suggested for other savanna mammals, further analyses of longer mtDNA sequences, ideally of full mtDNA genomes as well as of nuclear sequences from multiple independent genetic loci are necessary.

## CONCLUSION

Our study indicates the importance of dense taxon sampling for revealing the genetic diversity of African green monkeys. It also shows that mtDNA genomes of only few taxa do not effectively reflect the diversity of this species complex. The study of further mtDNA markers or complete mtDNA genomes as well as of nuclear DNA markers from a sample set that represents the diversity of African green monkeys can now be used to improve support of basal relationships and help to obtain a clearer picture of the phylogeography of African green monkeys.

Although our data set includes samples from most African green monkey taxa, subsequent studies should consider further samples from Nigeria, South Sudan, and Southeast Africa to provide additional information on species borders in those regions (question marks in Fig. 3B). In general, we found that the mtDNA diversity of African green monkeys does not conform to any of the suggested classifications and also that species distributions might need revisions. However, since we found evidence of introgressive hybridization in almost all contact zones between species, mtDNA diversity cannot be regarded as equivalent to species diversity and the analysis of maternally inherited markers alone is not appropriate to delimit species. Therefore, we do not consider any taxonomic changes here and advertise studies of nuclear markers to clarify the taxonomic status of the obtained mtDNA clades and the possible impact of hybridization on the mtDNA phylogeny of African green monkeys. Nevertheless, since our data present genetic evidence for the distinctiveness of *C. djamdjamensis* from the Bale Mountains NP and further confirm ongoing hybridization with *C. aethiops*, more attention should be paid to the conservation of this endemic species and to the protection of its restricted habitat.

## Supplementary material

Disclaimer: Supplementary materials have been peer-reviewed but not copyedited.

**Table SI** Taxon identity, sampling sites and GenBank accession numbers of the cytochrome *b* sequences of 126 *Chlorocebus* samples used in this study. Taxa are abbreviated with three initial letters and stars indicate type specimens.

**Table SII** Primer sequences, product sizes and annealing temperatures (T_a_) used for the amplification of the cytochrome *b* gene in this study.

**Figure S1.** Ranges of genetic distances (min, mean, max) within and among mtDNA clades in percent. Depicted are intra and inter-clade distances using a classification of (A) seven (C I-VII) and (B) nine (C1-9) mtDNA clades.
