# Supplementary material for: Mitochondrial Diversity and Distribution of African Green Monkeys (Chlorocebus Gray, 1870)
Source: Am J Primatol. 2013 Jan 10;75(4):350–60. doi: 10.1002/ajp.22113 (PMC3613741; doi:10.1002/ajp.22113)
Supplement: Supplementary file 1 [file ajp0075-0350-SD1.doc]

**Supplemental material**

**Table SI** Taxon identity, sampling sites and GenBank accession numbers of the cytochrome *b* sequences of 126 *Chlorocebus* samples used in this study. Taxa are abbreviated with three initial letters and stars indicate type specimens.

| **ID** | **Taxon** | **Country** | **Sample site** | **Longitude Latitude** | **Nature** | **Institution, Collection No.** | **GenBank**  **Cyt *b*** |
| --- | --- | --- | --- | --- | --- | --- | --- |
| I | pyg | Republic of South Africa | - | - |  | GenBank | EF597500 |
| II | pyg | Tanzania/  Kenya | - | - |  | GenBank | EF597501 |
| III | tan | Central African Republic | - | - |  | GenBank | EF597502 |
| IV | sab | Senegal | - | - |  | GenBank | EF597503 |
| 1 | aet | Eritrea | Fil Fil | 15.61720  38.97075 | hair | DPZ | JX983734 |
| 2 | aet | Eritrea | Fil Fil | 15.61720  38.97075 | hair | DPZ | JX983735 |
| 3 | aet | Eritrea | Faulena | 14.78750  37.99580 | hair | DPZ | JX983733 |
| 8 | aet | Eritrea | Anseba River | 15.70652  38.62617 | hair | DPZ | JX983730 |
| 9 | aet | Eritrea | Mt. Bizen | 15.33333  39.06194 | hair | Zoo Asmara | JX983731 |
| 11 | aet | Eritrea | Mt. Bizen | 15.33333  39.06194 | hair | Zoo Asmara | JX983732 |
| 26 | tan | Central African Republic | - | - | hair | Museum Besancon | JX983846 |
| 64 | pyg | Republic of South Africa | Loskop Dam NR | -25.42158  29.295536 | feces | DPZ | JX983769 |
| 65 | pyg | Republic of South Africa | Loskop Dam NR | -25.42158  29.295536 | feces | DPZ | JX983770 |
| 70 | cyn | Zambia | Kasanka NP | -12.57276  30.23332 | feces | DPZ | JX983756 |
| 72 | pyg | Zambia | Shiwa N’gandu | -11.19677  31.73892 | feces | DPZ | JX983752 |
| 75 | pyg | Republic of South Africa | Bedford | -32.68334  26.08350 | skin | ZFMK 61.269 | JX983771 |
| 78 | pyg | Republic of South Africa | Bedford | -32.68334  26.08350 | skin | ZFMK 62.3 | JX983772 |
| 91 | cyn | Zambia | Fibwe | -12.59050  30.25201 | feces | DPZ | JX983755 |
| 101 | cyn | Zambia | Chunga | -15.04440  25.99910 | feces | DPZ | JX983754 |
| 102 | tan | Nigeria | Gashaka Gumpi NP | 11.19677  31.73891 | feces | DPZ | JX983843 |
| 103 | tan | Nigeria | Gashaka Gumpi NP | 11.19677  31.73891 | feces | DPZ | JX983844 |
| 104 | tan | Nigeria | Gashaka Gumpi NP | 11.19677  31.73891 | feces | DPZ | JX983845 |
| 118 | sab | Senegal | Niokolo Koba NP | 13.07536  12.72239 | feces | DPZ | JX983827 |
| 120 | sab | Senegal | Niokolo Koba NP | 13.02577  13.23736 | feces | DPZ | JX983828 |
| 138 | sab | Ghana | Bui NP | 8.29083  -2.28465 | feces | DPZ | JX983804 |
| 139 | sab | Ghana | Bui NP | 8.29114  -2.28431 | feces | DPZ | JX983805 |
| 142 | sab | Ghana | Bui NP | 8.29114  -2.28431 | feces | DPZ | JX983806 |
| 143 | sab | Ghana | Bui NP | 8.29090  -2.28388 | feces | DPZ | JX983807 |
| 144 | sab | Ghana | Bui NP | 8.29126  -2.28315 | feces | DPZ | JX983808 |
| 145 | sab | Ghana | Bui NP | 8.29083  -2.28465 | feces | DPZ | JX983809 |
| 146 | sab | Ghana | Bui NP | 8.29083  -2.28465 | feces | DPZ | JX983810 |
| 153 | sab | Ghana | Mole NP | 9.26008  -1.86058 | feces | DPZ | JX983811 |
| 158 | sab | Ghana | Mole NP | 9.26058  -1.86147 | feces | DPZ | JX983812 |
| 160 | sab | Ghana | Mole NP | 9.25876  -1.84714 | feces | DPZ | JX983813 |
| 161 | sab | Ghana | Mole NP | 9.25876  -1.84714 | feces | DPZ | JX983814 |
| 165 | sab | Ghana | Mole NP | 9.25876  -1.84714 | feces | DPZ | JX983815 |
| 166 | sab | Ghana | Mole NP | 9.25183  -1.86105 | feces | DPZ | JX983816 |
| 167 | sab | Ghana | Mole NP | 9.25183  -1.86105 | feces | DPZ | JX983817 |
| 168 | sab | Ghana | Mole NP | 9.25183  -1.86105 | feces | DPZ | JX983818 |
| 169 | sab | Ghana | Mole NP | 9.25183  -1.86105 | feces | DPZ | JX983819 |
| 170 | sab | Ghana | Mole NP | 9.25183  -1.86105 | feces | DPZ | JX983820 |
| 171 | sab | Ghana | Mole NP | 9.25183  -1.86105 | feces | DPZ | JX983821 |
| 172 | sab | Ghana | Mole NP | 9.25183  -1.86105 | feces | DPZ | JX983822 |
| 191 | tan | Ghana | Kalapka RR | 6.45293  0.38055 | feces | DPZ | JX983839 |
| 192 | tan | Ghana | Kalapka RR | 6.45293  0.38055 | feces | DPZ | JX983840 |
| 193 | tan | Ghana | Kalapka RR | 6.45293  0.38055 | feces | DPZ | JX983841 |
| 203 | tan | Ghana | Shai Hills RR | 5.89777  0.06897 | feces | DPZ | JX983823 |
| 208 | tan | Ghana | Shai Hills RR | 5.89001  0.04382 | feces | DPZ | JX983824 |
| 212 | tan | Burkina Faso | Buffle Rouge | 11.30183  1.04397 | feces | DPZ | JX983836 |
| 213 | tan | Burkina Faso | Buffle Rouge | 11.30183  1.04397 | feces | DPZ | JX983837 |
| 215 | tan | Burkina Faso | Buffle Rouge | 11.30183  1.04397 | feces | DPZ | JX983838 |
| 223 | tan | Burkina Faso | Park D’Arly | 11.60094  1.39187 | feces | DPZ | JX983830 |
| 224 | tan | Burkina Faso | Park D’Arly | 11.60094  1.39187 | feces | DPZ | JX983831 |
| 225 | tan | Burkina Faso | Park D’Arly | 11.60094  1.39187 | feces | DPZ | JX983832 |
| 226 | tan | Burkina Faso | Park D’Arly | 11.58281  1.46103 | feces | DPZ | JX983833 |
| 227 | tan | Burkina Faso | Park D’Arly | 11.58281  1.46103 | feces | DPZ | JX983834 |
| 228 | tan | Burkina Faso | Park D’Arly | 11.58087  1.45781 | feces | DPZ | JX983835 |
| 233 | tan | Burkina Faso | Park D’Arly | 11.58087  1.45781 | feces | DPZ | JX983829 |
| 239 | sab | Burkina Faso | Ranch Nazinga | 11.16182  -1.60967 | feces | DPZ | JX983793 |
| 241 | sab | Burkina Faso | Ranch Nazinga | 11.13514  -1.61265 | feces | DPZ | JX983794 |
| 246 | sab | Burkina Faso | Ranch Nazinga | 11.15594  -1.61445 | feces | DPZ | JX983795 |
| 247 | sab | Burkina Faso | Ranch Nazinga | 11.14870  -1.62334 | feces | DPZ | JX983796 |
| 248 | sab | Burkina Faso | Ranch Nazinga | 11.14870  -1.62334 | feces | DPZ | JX983797 |
| 250 | sab | Burkina Faso | Ranch Nazinga | 11.14870  -1.62334 | feces | DPZ | JX983798 |
| 251 | sab | Burkina Faso | Ranch Nazinga | 11.14870  -1.62334 | feces | DPZ | JX983799 |
| 254 | sab | Burkina Faso | Ranch Nazinga | 11.15547  -1.61030 | feces | DPZ | JX983800 |
| 255 | sab | Burkina Faso | Ranch Nazinga | 11.15547  -1.61030 | feces | DPZ | JX983801 |
| 256 | sab | Burkina Faso | Ranch Nazinga | 11.15547  -1.61030 | feces | DPZ | JX983802 |
| 264 | sab | Burkina Faso | FC Deux Bale | 11.55079  -2.95757 | feces | DPZ | JX983792 |
| 268 | sab | Burkina Faso | Comoe Leraba NP | 9.90260  -4.65454 | feces | DPZ | JX983778 |
| 269 | sab | Burkina Faso | Comoe Leraba NP | 9.90260  -4.65454 | feces | DPZ | JX983779 |
| 271 | sab | Burkina Faso | Comoe Leraba NP | 9.90260  -4.65454 | feces | DPZ | JX983780 |
| 272 | sab | Burkina Faso | Comoe Leraba NP | 9.84896  -4.62302 | feces | DPZ | JX983781 |
| 273 | sab | Burkina Faso | Comoe Leraba NP | 9.84896  -4.62302 | feces | DPZ | JX983782 |
| 275 | sab | Burkina Faso | Comoe Leraba NP | 9.84896  -4.62302 | feces | DPZ | JX983783 |
| 277 | sab | Burkina Faso | Comoe Leraba NP | 9.85291  -4.61415 | feces | DPZ | JX983784 |
| 279 | sab | Burkina Faso | Comoe Leraba NP | 9.86992  -4.65626 | feces | DPZ | JX983785 |
| 281 | sab | Burkina Faso | Comoe Leraba NP | 9.86992  -4.65626 | feces | DPZ | JX983786 |
| 283 | sab | Burkina Faso | Comoe Leraba NP | 9.86207  -4.66974 | feces | DPZ | JX983787 |
| 284 | sab | Burkina Faso | Comoe Leraba NP | 9.86207  -4.66974 | feces | DPZ | JX983788 |
| 285 | sab | Burkina Faso | Comoe Leraba NP | 9.86207  -4.66974 | feces | DPZ | JX983789 |
| 286 | sab | Burkina Faso | Comoe Leraba NP | 9.86207  -4.66974 | feces | DPZ | JX983790 |
| 293 | sab | Burkina Faso | Comoe Leraba NP | 9.77530  4.60271 | feces | DPZ | JX983791 |
| 316 | aet | Ethiopia | Tana | 11.68854  37.32800 | feces | DPZ | JX983749 |
| 317 | aet | Ethiopia | Tana | 11.68854  37.32800 | feces | DPZ | JX983750 |
| 319 | dja | Ethiopia | Harenna, Bale Mts. NP | 6.75993  39.73711 | feces | DPZ | JX983758 |
| 320 | dja | Ethiopia | Harenna, Bale Mts. NP | 6.75993  39.73711 | feces | DPZ | JX983759 |
| 323 | dja | Ethiopia | Harenna, Bale Mts. NP | 6.75993  39.73711 | feces | DPZ | JX983760 |
| 330 | aet | Ethiopia | Lake Awassa | 7.04809  38.46210 | feces | DPZ | JX983741 |
| 331 | aet | Ethiopia | Lake Awassa | 7.04809  38.46210 | feces | DPZ | JX983742 |
| 373 | aet | Ethiopia | Sodore | 8.40321  39.39247 | feces | DPZ | JX983740 |
| 391 | aet/pyg | Ethiopia | Arba Minch | 6.03850  37.57189 | feces | DPZ | JX983739 |
| 400 | aet | Ethiopia | Jinka | 5.72704  36.64093 | feces | DPZ | JX983746 |
| 421 | pyg | Ethiopia | Yabello | 4.89596  38.07043 | feces | DPZ | JX983766 |
| 433 | dja/aet | Ethiopia | Bubbe Kersa | 6.14111  38.73954 | feces | DPZ | JX983761 |
| 436 | dja/aet | Ethiopia | Gossa | 6.28349  38.67784 | feces | DPZ | JX983762 |
| 438 | aet | Ethiopia | Jimma | 7.69243  36.80676 | feces | DPZ | JX983744 |
| 439 | aet | Ethiopia | Jimma | 7.69243  36.80676 | feces | DPZ | JX983745 |
| 451 | aet | Ethiopia | Bonga | 7.26645  36.23131 | feces | DPZ | JX983743 |
| 464 | aet | Ethiopia | Woliso | 8.53230  37.98187 | feces | DPZ | JX983751 |
| 484 | aet | Ethiopia | Menegasha | 8.96569  38.52645 | feces | DPZ | JX983747 |
| 485 | aet | Ethiopia | Menegasha | 8.96569  38.52645 | feces | DPZ | JX983748 |
| 496 | pyg | Ethiopia | Sof Omar | 6.91205  40.84583 | feces | DPZ | JX983764 |
| 503 | pyg | Tanzania | Iringa | -7.279220  35.738340 | feces | DPZ | JX983773 |
| 504 | pyg | Kenya | Sukari Ranch | -1.25000  37.10000 | skin | NMK SUK3 | JX983767 |
| 507 | pyg | Kenya | Charangani | 0.98333  35.21667 | skin | NMK 4836 | JX983851 |
| 513 | pyg | Kenya | Kilgoris | -1.00541  34.87197 | skin | NMK 6783 | JX983850 |
| 520 | sab | Liberia | Gola Country | 7.44317  -10.77779 | skin | ZSM 5 | JX983825 |
| 522 | pyg | Tanzania | Fundo Island, Pemba | -5.05264  39.64780 | tissue | MfN 26 | JX983774 |
| 524 | pyg | Tanzania | Bukoba | -1.32404  31.80739 | skin | MfN 9091 | JX983775 |
| 528 | ell* | Ethiopia | Suksuk River | 7.78830  38.67257 | skin | MfN 35504 | JX983736 |
| 529 | dja* | Ethiopia | Abera, 3200m | 6.44189  38.47116 | skin | MfN 35505 | JX983757 |
| 530 | mat* | Ethiopia | Malo(Naja), Omo River | 6.58333  36.55000 | tissue | MfN 35509 | JX983737 |
| 535 | sab | Mauretania | Podor, Senegal River | 16.54844  -14.24304 | tooth | MfN 40413 | JX983826 |
| 536 | tan | Togo | Bassari | 9.23291  0.76747 | skin | MfN 77410 | JX983842 |
| 539 | tan | Cameroon | Dschang District | 5.39621  9.87775 | tooth | MfN 87395 | JX983848 |
| 540 | pyg | Kenya | Guaso Njiro River | 0.31035  37.19262 | tissue | MfN 87411 | JX983768 |
| 545 | pyg | Somalia | Bardera | 2.33364  42.28266 | tissue | MfN 87445 | JX983765 |
| 546 | aet | Ethiopia | Roba Butta | 6.81667  40.76667 | tooth | MfN 87449 | JX983763 |
| 549 | pyg | Tanzania | Mkulwe | -8.58079  32.31928 | tooth | MfN 87461 | JX983776 |
| 553 | sab | Ghana | Kratshi/Krachi | 7.80000  -0.05000 | tissue | MfN 87496 | JX983803 |
| 560 | pyg | Uganda | W of Lake Albert | 2.21534  31.28184 | tissue | MfN 87511 | JX983849 |
| 561 | tan | Cameroon | Lake Chad | 12.94512  14.33129 | tissue | MfN 87512 | JX983847 |
| 563 | pyg | Tanzania | Pangani River, middle course | -5.16886  38.35409 | tissue | MfN 87557 | JX983777 |
| 566 | cyn | Angola | Cubal River, Benguela | -12.81594  13.65009 | tooth | MfN 87998 | JX983753 |
| 567 | ell* | Ethiopia | Suksuk River | 7.78830  38.67257 | tissue | MfN 88754 | JX983738 |

NP= National Park, NR= Nature Reserve, FC= Forêt Classée, RR= Resource Reserve.

DPZ= Deutsches Primatenzentrum, Goettingen, Germany

ZFMK= Zoologisches Forschungsmuseum Alexander Koenig, Bonn, Germany

NMK= National Museums of Kenya, Nairobi, Kenya

ZSM= Zoologische Staatssammlung München, Munich, Germany

MfN= Museum für Naturkunde, Berlin, Germany

aet = *aethiops*, cyn = *cynosuros*, dja = *djamdjamensis*, ell = *ellenbecki*, mat = *matschiei*, pyg =*pygerythrus*, sab =*sabaeus*, tan = *tantalus*

**Table SII** Primer sequences, product sizes and annealing temperatures (T_a_) used for the amplification of the cytochrome *b* gene in this study.

| Primer ID | Sequence (5’-3’) | Size [bp] | T_a_ [°C] |
| --- | --- | --- | --- |
| 2 overlapping fragments | | | |
| cytbAfw | CCACCGTTGTACTTCAACTAC | 681 | 62 |
| cytbArv | TTGTCTGAGTCTGATGAGATTC |  | 62 |
| cytbBfw | CCACCCTTTCACGATTCTTCA | 664 | 62 |
| cytbBrv | TAGTTTACAAGACTAGTGTATTAG |  | 62 |
| 4 overlapping fragments | | | |
| cytbA1fw | CCACCGTTGTACTTCAACTAC | 369 | 62 |
| cytbA1rv | CAGGTTTTTAGGAGAAGGAATG |  | 62 |
| cytbA2fw | GGCGCCTCCATATTTTTCATC | 334 | 62 |
| cytbA2rv | TTGTCTGAGTCTGATGAGATTC |  | 62 |
| cytbB1fw | CCACCCTTTCACGATTCTTCA | 382 | 62 |
| cytbB1rv | ATGAGGATTGATAGGAAGAGTG |  | 62 |
| cytbB2fw | CCCTCCACACATCAAACCAG | 390 | 62 |
| cytbB2rv | TAGTTTACAAGACTAGTGTATTAG |  | 62 |
| cytbB2fw4 | YCCACACATCAAACCAG | 322 | 52 |
| cytbB2rv4* | TAGAATGCCAGTTTTGGG |  | 52 |
| 6 overlapping fragments | | | |
| cytbM1fw | CCACCGTTGTACTTCAACTAC | 269 | 62 |
| cytbM1rv | CGAATGATTCAGCCGTGGTTT |  | 62 |
| cytbM2fw | CAGACACCTCTTCTGCCTTC | 262 | 62 |
| cytbM2rv | GTTGCYCCYCAGAATGATATT |  | 62 |
| cytbM3fw | ATAGCAACAGCYTTYATAGGCT | 252 | 62 |
| cytbM3rv | GTGTAGAAACAGCAGATGGAC |  | 62 |
| cytbM4fw | CGATTCTTCACCCTACACTTTA | 283 | 62 |
| cytbM4rv | CTGGTTTGATGTGTGGRGGG |  | 62 |
| cytbM5fw | CTRAACGACCCAGACAACTA | 272 | 62 |
| cytbM5rv | CTTCCGATCCAGGTGAGGG |  | 62 |
| cytbM6fw | AGCATAATATTCCGCCCACTTA | 242 | 62 |
| cytbM6rv | TAGTTTACAAGACTAGTGTATTAG |  | 62 |

* taken from Naidu et al. 2011

**Fig. S1**


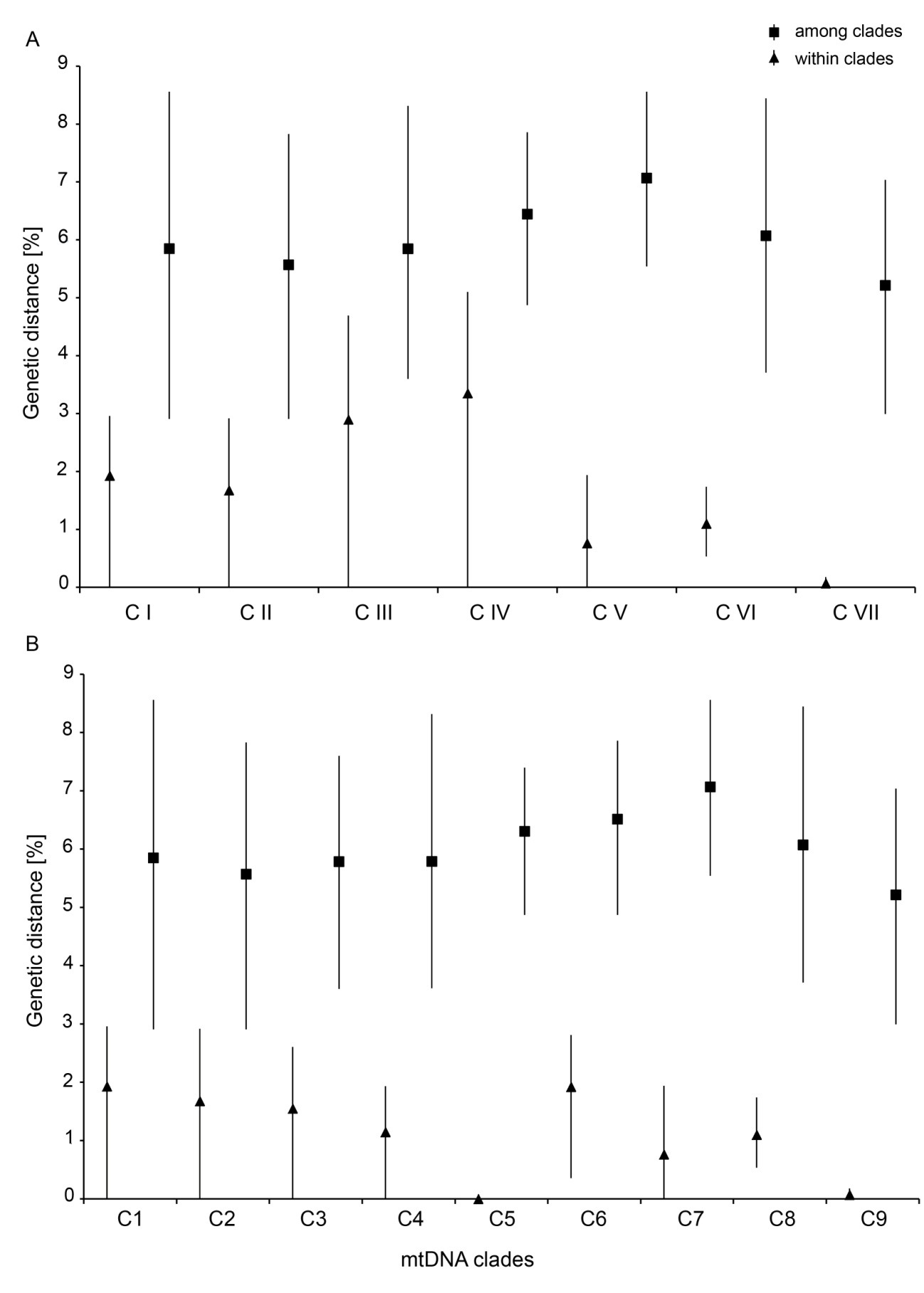


Figure S1. Ranges of genetic distances (min, mean, max) within and among mtDNA clades in percent. Depicted are intra and inter-clade distances using a classification of (A) seven (C I-VII) and (B) nine (C1-9) mtDNA clades.
